# BMP2–ERK–ATF4 Axis-Based 6-methoxybenzofuran Compound I-9 Acts as Candidate Drug for Bone Formation and Anti-Osteoporosis

**DOI:** 10.3390/ijms25063350

**Published:** 2024-03-15

**Authors:** Ziying Zhou, Chenxi Zhao, Siyan Li, Xiaoyang Han, Jiangyi Zhu, Situ Xue, Zhuorong Li

**Affiliations:** Institute of Medicinal Biotechnology, Chinese Academy of Medical Sciences & Peking Union Medical College, Beijing 100050, China; zhouziying202108@163.com (Z.Z.); zhaochenxi19920220@163.com (C.Z.); lisiyan990321@163.com (S.L.); 15305191141@163.com (X.H.); 13605132482@163.com (J.Z.)

**Keywords:** osteoporosis, 6-methoxybenzofuran, ATF4, BMP-2, bone formation, osteoblast

## Abstract

As the global population ages, the number of patients with osteoporosis is rapidly rising. The existing first-line clinical drugs are bone resorption inhibitors that have difficulty restoring the bone mass of elderly patients to the safe range. The range and period of use of existing peptides and monoclonal antibodies are limited, and small-molecule bone formation–promoting drugs are urgently required. We established an I-9 synthesis route with high yield, simple operation, and low cost that was suitable for future large-scale production. I-9 administration promoted bone formation and increased bone mass in mice with low bone mass in an aged C57 mouse model. Our findings revealed a hitherto undescribed pathway involving the BMP2–ERK–ATF4 axis that promotes osteoblast differentiation; I-9 has favorable biosafety in mice. This study systematically investigated the efficacy, safety, and mechanism of I-9 for treating osteoporosis and positions this drug for preclinical research in the future. Thus, this study has promoted the development of small-molecule bone-promoting drugs.

## 1. Introduction

Osteoporosis is a common skeletal disease characterized by low bone mass, bone tissue microstructure degradation, increased bone fragility, and fracture susceptibility [1]. Osteoporosis can occur at any age but is more common in postmenopausal women and elderly men. According to the etiology, osteoporosis is divided into two categories: primary and secondary. Primary osteoporosis mainly includes postmenopausal osteoporosis (type I) and senior osteoporosis (type II), which account for approximately 90% of cases [2,3]. The prevalence of osteoporosis in Asia, Europe, and the USA is 24.3%, 16.7%, and 11.5%, respectively [4,5,6,7]. Osteoporosis can cause pain, kyphosis, respiratory function impairment, and bone fracture; osteoporotic fracture is a main important cause of disability and even death in the elderly. Hip fractures are the most severe among all fractures. Within one year after a hip fracture, 20% of patients may die from various complications, and approximately 50% of patients are disabled and their quality of life significantly decreased [8,9,10]. The main consequences of spinal fractures are back pain, kyphosis, and decreased height, which negatively affect physical function, self-esteem, body image, and emotional well-being [11]. The medical treatment and care of fractures can also place a heavy burden on families and society. The ageing society and the high correlation between osteoporosis and age make this disease an increasingly important public health problem.

Anti-resorption drugs remain the most common drugs used for treating osteoporosis [12,13,14,15]. Bisphosphonates, mainly ibandronate, risedronate, alendronate, and zoledronate, are the first-line drugs for most patients because of factors such as effectiveness, safety, and affordability [16]. A large number of patients seek medical treatment late because of their low awareness rate, and their bone mass is consequently seriously insufficient at the beginning of treatment. Although bone resorption inhibitors can prevent further bone loss, they cannot reverse the bone loss that has already occurred. Compared with bone resorption inhibitors, drugs that promote bone formation have significantly better effects in increasing bone density and have stronger anti-fracture effects [17]. Parathyroid hormone analogs [18,19,20,21,22] and Romosozumb [23] are bone-forming agents whose clinical use is limited to 24 and 12 months, respectively. The mainstream bone formation–promoting drugs currently used in clinical practice are peptides or monoclonal antibodies, which can only be administered by injection, and patients have poor tolerance to long-term medication. Furthermore, the production process of these drugs is complex and expensive. Thus, anti-osteoporosis drugs remain a major unmet medical need, and the development of small-molecule drugs to promote bone formation is critical to meet this need.

**Figure 1:**
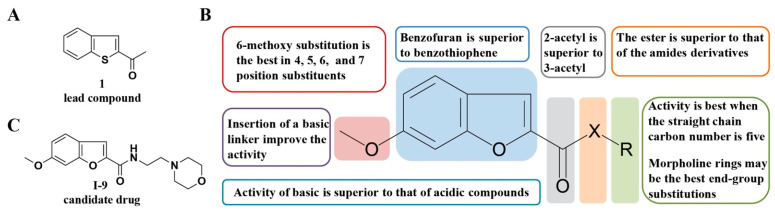
Study on SAR of 6-methoxybenzofuran derivatives against osteoporosis: (**A**) Structure of lead compound 1. (**B**) Summary of SAR of 6-methoxybenzofuran derivatives. (**C**) Structure of I-9.

In recent years, drug targets have been explored that can induce bone mass increase and bone structure recovery by stimulating osteoblast activity. Bone morphogenetic protein (BMP) is a multifunctional growth factor that belongs to the transforming growth factor β (TGF-β) superfamily. Bone Morphogenetic protein 2 (BMP2) is responsible for inducing endochondral and periosteal ossification and activating fat and cartilage generation signaling pathways [24]. In 2002, the FDA approved the use of recombinant human BMP2 in spinal fusion surgery, tibial shaft repair, and maxillary sinus reconstruction surgery [25]. In our previous work, we established a high-throughput screening model based on *Bmp2* and found that 6-methoxy-benzofuran compounds can upregulate *Bmp2* expression and lead compounds (Figure 1A) can promote bone formation with a clear anti-osteoporotic activity [26,27,28]. We systematically investigated 6-methoxybenzofuran derivatives as potential candidates for senior osteoporosis (SOP) treatment in senile osteoporosis. First, the therapeutic and prophylactic effects of 125 on SOP were evaluated in aged C57 mouse models and SAMP-6 mice. Subsequently, RNA-SEQ analysis was performed to elucidate the underlying mechanism. We systematically studied the structure–activity relationship (SAR) of these compounds (Figure 1B), and found that I-9 (Figure 1C) may have the best activity of promoting bone formation and anti-osteoporosis [29].

In this study, we used I-9 as a candidate drug to study the synthesis methodology, efficacy, and mechanism. First, the key steps in I-9 synthesis were studied to optimize the I-9 synthesis route. The pharmacodynamics of I-9 were then evaluated in naturally aged C57 mice. The vertebral density of live mice was quantitatively measured via micro-computed tomography (micro-CT) after 0–4 months of treatment. Then, the morphology of the isolated femur was quantitatively evaluated. The effect of I-9 on the number of femoral osteoblasts was quantitatively studied by section staining. Finally, the mechanism of I-9 promoting bone formation was investigated. We examined the effect of I-9 on MAPK pathway-related gene expression in mouse liver and bone tissue. We further studied the relationship between activating transcription factor 4 (ATF4) and the extracellular signal-regulated kinase (ERK) signaling pathway and osteoporosis by using mouse embryonic osteoblastic precursor cells (MC3T3-E1).

## 2. Results

### 2.1. Optimization of I-9 Synthesis

**Scheme 1:**
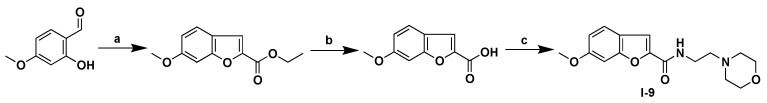
Synthesis of I-9 and 125 ^a^. ^a^ Reagents and conditions: (**a**) BrCH_2_COOC_2_H_5_, DMF, anhydrous K_2_CO_3_, 0 °C, 1 h, 90 °C, 18 h; (**b**) 0.1 N NaOH, H_2_O, dioxane, 40 °C, 1 h; (**c**) HOBt, EDCI, DCM, r.t, 16 h.

Scheme 1 describes the synthetic pathways of targets I-9. Using 2-hydroxy-4-methoxybenzaldehyde and ethyl bromoacetate as raw materials, I-9 was obtained by aldol condensation, hydrolysis, and condensation. To explore the conditions of large-scale synthesis of I-9, methodological research was conducted on step c (Table 1). We tried different condensation agents and reaction conditions to synthesize the amide. When other conditions were consistent, 1-Hydroxybenzotriazole (HOBt) and 1-Ethyl-3-(3-dimethylaminopropyl) carbodiimide hydrochloride (EDCI) were used as catalytic condensation agents, and the I-9 yield was the highest. The urea produced after the EDCI reaction was water soluble and easy to wash, and the reaction condition was warm. HOBt and EDCI are cheap and easy to obtain and, therefore, suitable for mass synthesis.

### 2.2. Effect of I-9 on Bone Mineral Density in Aged C57 Mice

**Figure 2:**
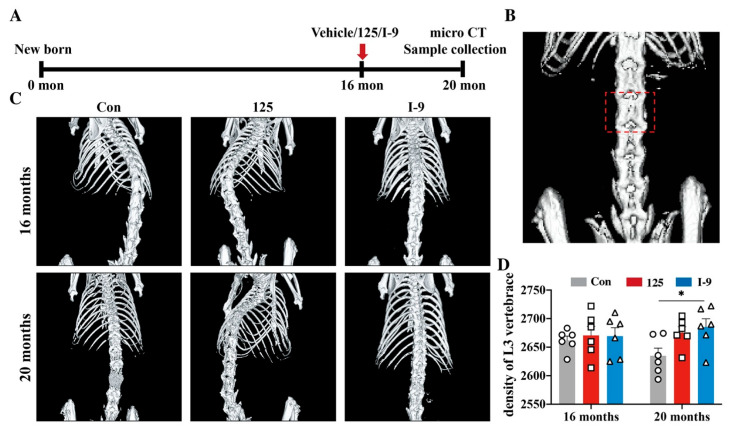
Micro-CT of C57 aged mice. C57 mice at 16 months of age were injected with 125 and I-9 (30 mg/kg/day, 6 days a week), and the BMD of the third vertebra (L3) was measured before and 4 months after administration: (**A**) Diagram of administration of I-9 and 125 in mice. (**B**) Schematic diagram of the mouse L3 as a region of interest, and the red rectangle represents the quantitative area. (**C**) Representative images of the thoracic vertebrae and vertebrae of mice were reconstructed using micro-CT before and after 4 months of treatment. (**D**) L3 BMD in the senile control group, senile 125 group, and senile I-9 group before and 4 months after treatment (n = 6 per group). Data are expressed as mean ± SEM. One-way analysis of variance was used to compare groups. * *p* < 0.05.

To evaluate the anti-osteoporotic effects of I-9 in vivo, we selected 16-month-old C57 mice that were postmenopausal at approximately 12 months of age, at which time secondary bone loss had occurred and bone deficiency was severe. Previously, we found 125 (N-(2-(dimethylamino)ethyl)-6-methoxybenzofuran-2-carboxamide methanesulfonate) exact anti-osteoporosis effects on promoting bone formation. In this study, we used 125 as a positive-control drug and administered I-9 and 125 to C57 mice for a total of 4 months. C57 mice were measured in vivo using micro-CT before and after 4 months of administration of 125 and I-9, and three-dimensional models were established (Figure 2A). The third vertebral body (L3) of mice was selected as the region of interest (Figure 2B), and the bone mineral density (BMD) was quantitatively analyzed by using the built-in software (Analyze 2.0). The CT image closest to the average BMD (L3) in each group was selected as the display image (Figure 2C). The images of each group were compared and showed that bone porosity in the control (Con) group increased after 4 months of administration, while bone porosity in the I-9 and 125 groups was lower than that in the Con group, whereas bone status was better than that in the Con group. Quantitative results are shown in Figure 2D. The average grey value of bone mineral density decreased slightly in the Con group at 16 to 20 months of age. The L3 vertebral BMD in the 125 group remained unchanged at 16 to 20 months, whereas the BMD in the I-9 group significantly increased at 16 to 20 months, and a significant difference in BMD was present between the I-9 and Con groups at 20 months of age. These results indicated that I-9 upregulated vertebral bone density and improved the whole-body bone status of aged C57 mice.

### 2.3. Effect of I-9 on Femur Histomorphometry in Aged C57 Mice

**Figure 3:**
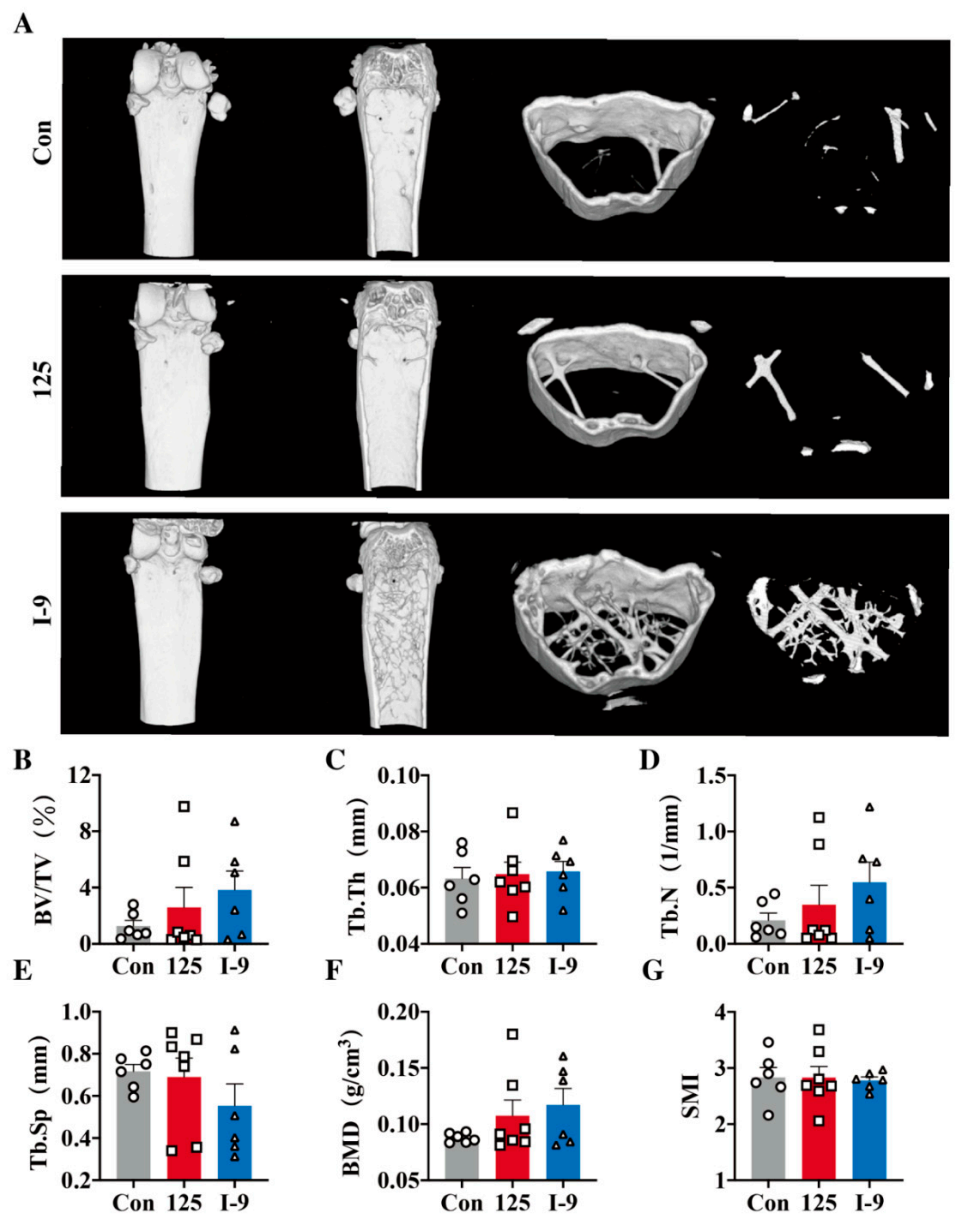
Isolated micro-CT of the femur in aged C57 mice: (**A**) From top to bottom, the overall 3D image of the femur scanning area is shown for the Con, 125, and I-9 groups. Three-dimensional maps of the area of interest from left to right: coronal view of the scanning area of femur; coronal-sectional view; cross-section view, 45° backward (shows the thickness of the analysis area); and bone trabeculae in the area of interest (cross-section view); (**B**) Quantitative micro-CT analysis of bone volume fraction (BV/TV, %) of the right femur in mice; (**C**) bone trabecular thickness (Tb.Th, mm); (**D**) Number of bone trabeculae (Tb.N, 1/mm); (**E**) trabecular separation (Tb.Sp, mm); (**F**) Structural Model Index (SMI); (**G**) Bone density (BMD, g/cm^3^) (n = 6–7). Data are expressed as mean ± SEM.

After 4 months of administration, the right femur of each group of mice was excised for bone morphology study, and the transverse section and coronal plane of the femur were reconstructed by micro-CT scanning. The mouse femur closest to the mean BT/TV value of each group was selected for the diagram (Figure 3A). In the Con group, the femoral bone cortex became thinner, and the number of bone trabeculae decreased sharply. After the administration of 125 and I-9, the bone in the scan area of the femur was more compact, the number of bone trabeculae increased, the thickness increased, and the space between bone trabeculae was smaller; furthermore, the effect of I-9 was more apparent than that of 125. Quantitative bone morphology analysis confirmed that the mean bone volume fraction (BV/TV) of 125 and I-9 femur was 2.59% and 3.83%, respectively, which was 1.32% and 2.56% higher than that of the Con group (1.27%) (Figure 3B). Trabecular thickness (Tb.Th) was the average trabecular thickness, and no significant change occurred between the I-9 and 125 groups and the Con group (Figure 3C). The bone trabecular numbers (Tb.N) in 125 and I-9 were 0.35 and 0.55 per mm, respectively, and were increased by 0.14 (66.67%) and 0.34 (161.90%) compared with that of the Con group (0.21/mm) (Figure 3D). The trabecular distance (Tb.Sp) refers to the average width of the trabecular pulp cavity. The Tb.Sp of the I-9 group was 0.55 mm, which was reduced by 0.17 mm (23.61%) compared with that of the Con group (0.72 mm), where the Tb.Sp of the 125 group did not change significantly (Figure 3E). The structural model index (SMI) reflects the characteristics of a trabecular plate structure or rod-like structure. When osteoporosis occurs, trabecular bone changes from a plate structure to a rod-like structure, and the SMI value increases. I-9 and 125 had no significant effect on femur SMI in mice (Figure 3F). The BMD of 125 and I-9 was 0.108 and 0.117 g/cm^3^, respectively, and was significantly higher in the I-9 group than that in the Con group and slightly higher than that in the 125 group (Figure 3G). Thus, I-9 significantly improved the femur status of elderly C57 mice, and the effect was better than that of 125.

### 2.4. I-9 Increased the Number of Osteoblasts in Aged C57 Mice

**Figure 4:**
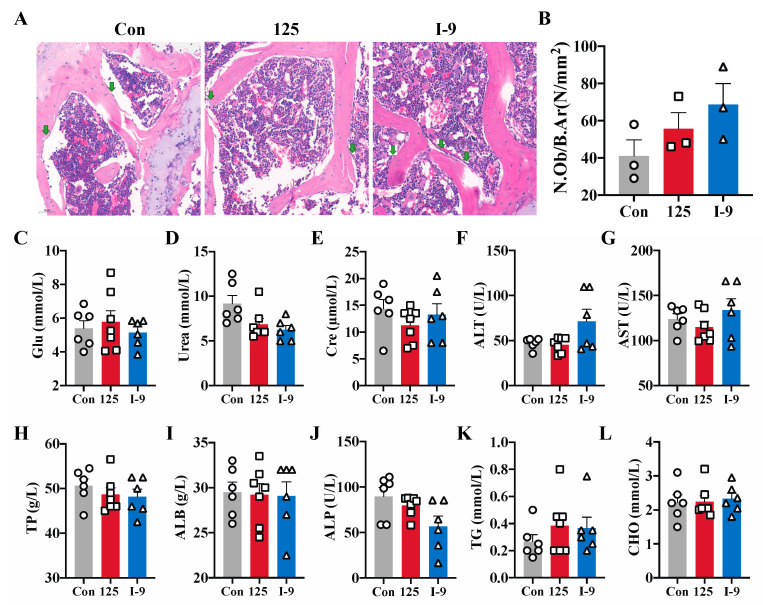
Staining of femur section and blood biochemical indexes of C57 mice after 4 months of I-9 treatment: (**A**) Representative images of the femur with hematoxylin–eosin staining. The green arrow indicates fusiform osteoblasts. Scale: 50 μm. (**B**) Number of osteoblasts per mm^2^ of trabecular bone surface (BS) (n = 3). Assessment of liver and kidney function in mice: glucose (Glu) (**C**), urea nitrogen (Urea) (**D**), creatinine (Cre) (**E**), alanine transaminase (ALT) (**F**), glutamic oxalate aminotransferase (AST) (**G**), total protein (TP) (**H**), albumin (ALB) (**I**), alkaline phosphatase (ALP) (**J**), triglyceride (TG) (**K**), and cholesterol (CHO) (**L**) (n = 6–7). Data are expressed as mean ± SEM.

To investigate the possible bone-promoting effect of I-9 in senile osteoporosis, hematoxylin and eosin staining was performed on the left femur of mice after 4 months of administration. Osteoblasts were clearly visible at 20× magnification (Figure 4A, green arrow). The cell bodies of osteoblasts stained by hematoxylin–eosin staining are large, often oblong or irregular, often distributed in clusters, sometimes single, with clear or fuzzy cell edges. The nucleus is elliptical or round, often on one side of the cell, in a coarse network, with 1 to 3 clear blue nucleolar-cytoplasm-rich, dark blue or light blue, often vacuole, and often elliptical light-stained areas farther from the nucleus. The mean number of osteoblasts was significantly higher in the I-9 group (68.67 per mm^2^) than in the Con group (41 per mm^2^) and was also slightly higher than that in the 125 group (55.67 per mm^2^). Thus, I-9 can upregulate the number of femoral osteoblasts in aged C57 mice (Figure 4B).

### 2.5. Long-Term Administration of I-9 Was Safe in Aged C57 Mice

Mouse behavior was observed twice a week over the four months of continuous administration. The mice in each group had a strong appetite, keen eyesight, rapid reaction, smooth body hair, slight licking phenomenon, and black wheat-like feces, indicating that I-9 had good biosafety. During the administration of the drug for four consecutive months, the mice showed no obvious weight loss, and no large number of animal deaths or tumor occurrence. After 4 months of administration, the serum of mice in each group was used for blood biochemical detection, including liver function index, kidney function index, blood glucose, lipid index, and other indicators. Biochemical indicators related to liver function include alanine transaminase, glutamic oxalate aminotransferase, alkaline phosphatase, albumin, and total protein. The renal function test evaluates the filtration function of the glomeruli and the excretion and absorption function of the renal tubules. Biochemical indicators related to renal function include urea nitrogen and creatinine. The related indexes of blood lipids were cholesterol and triglyceride. Mouse sera was taken for blood biochemical examination (Figure 4C–L). Long-term administration of I-9 had no significant effect on blood biochemical indexes of C57 mice, and all indexes were within the normal range. 

### 2.6. Benzofuran Derivatives Are Involved in the Regulation of the ERK Signaling Pathway in the Femur of Aged C57 Mice

**Figure 5:**
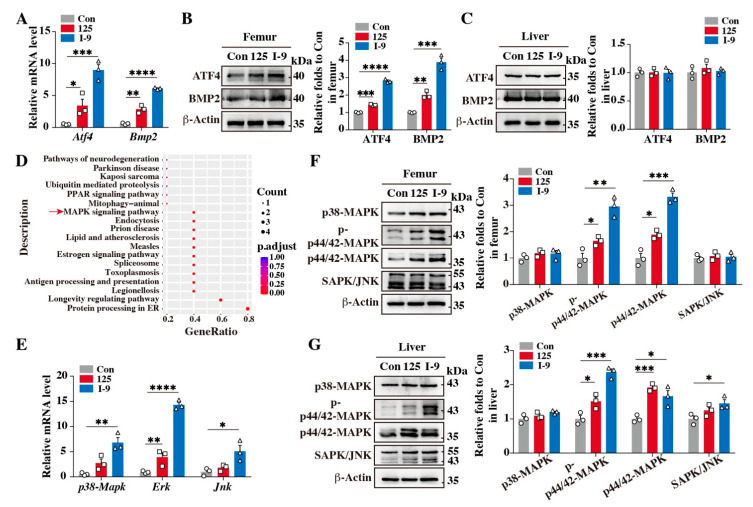
I-9 may activate ERK signaling to prevent osteoporosis: (**A**) RT–qPCR analysis of *Atf4* and *Bmp2* transcription in femur tissues from aged mice treated with Con, 125, or I-9 (n = 3); (**B**) Western blot showing ATF4 and BMP2 expression in femur tissues from aged mice treated with Con, 125, or I-9. Data are representative of three independent experiments; (**C**) Western blot showing ATF4 and BMP2 expression in liver tissues from aged mice treated with Con, 125 or I-9. Data are representative of three independent experiments; (**D**) KEGG pathway analysis illustrating increased expression in signaling pathways in chondroblasts following treatment with 125 and the red arrow indicating the MAPK signaling pathway; (**E**) RT–qPCR analysis of *p38-Mapk*, *Erk*, and *Jnk* transcription in femur tissues from aged mice treated with Con, 125 or I-9 (n = 3); (**F**) Western blot showing MAPK family protein expression in femur tissues from aged mice treated with Con, 125, or I-9. Data are representative of three independent experiments; (**G**) Western blot showing MAPK family protein expression in liver tissues from aged mice treated with Con, 125, or I-9. Three independent experiments in Con, 125 and I-9 group were indicated by circular symbols, squared symbols and triangular symbols, respectively. Data are presented as the mean ± SEM. Student’s *t*-tests were used to determine statistical significance; * *p* < 0.05, ** *p* < 0.01, *** *p* < 0.001, **** *p* < 0.0001.

In previous studies, we used single-cell sequencing experiments to find that benzofuran compounds could regulate the differential expression of genes in femoral osteoblasts of aged C57 mice. ERK activates RSK2 and phosphorylates ATF4 to regulate the formation of osteoblasts [30]. Irisin treatment of osteoporosis promotes the proliferation and differentiation of osteoblasts by activating the p38/ERK MAPK signaling pathway [31]. ERK inhibitor PD98059 inhibits the increase in COL1A1 gene expression induced by collagen hydrolysate (CH), indicating that the ERK signaling pathway mediates CH osteogenic activity [32]. The transcription factor *Atf4* was most closely related to osteoblast differentiation and bone tissue formation. As expected, the mRNA level of *Atf4* and *Bmp2* in the femur tissue of aged C57 mice was increased by treatment with 125 and further increased by treatment with I-9 (Figure 5A), as was the protein level in femur tissue of aged C57 mice instead of liver tissue (Figure 5B,C). The mitogen-activated protein kinase (MAPK) pathway has been reported to be closely related to osteoporosis, and ATF4 is involved in the regulation of osteoblast differentiation by the MAPK signaling pathway. MAPK signaling includes ERK1/2, p38, JNK, ERK5, and other components [33]. Activated ERK, p38, and JNK can enter the nucleus, activate downstream transcription factors, and regulate the proliferation, differentiation, and migration of osteoblasts [34]. To characterize the mechanism of 125 and I-9 in the treatment of OP, we evaluated the expression of MAPK family proteins. We used KEGG to analyze genes whose expression was increased in chondroblasts following treatment with 125. The MAPK signaling pathway was significantly increased in the 125-treatment group in comparison with that in the Con group (Figure 5D). The relative mRNA level of *Erk* was increased in the femur tissues of aged mice treated with 125 and was further increased in aged mice treated with I-9 (Figure 5E). The protein level of ERK1/2 and p-ERK1/2 was significantly improved by the treatment with 125 and I-9 in both femur and liver tissue; however, the p38 and JNK protein levels were not increased in the femur or liver tissues of aged mice treated with 125 or I-9, indicating that the ERK signaling pathway may be associated with the function of 125 and I-9 in osteoporosis (Figure 5F,G).

### 2.7. Benzofuran Derivatives May Upregulate ATF4 Expression by Increasing the p-ERK Protein Level to Promote Osteoblast Differentiation

**Figure 6:**
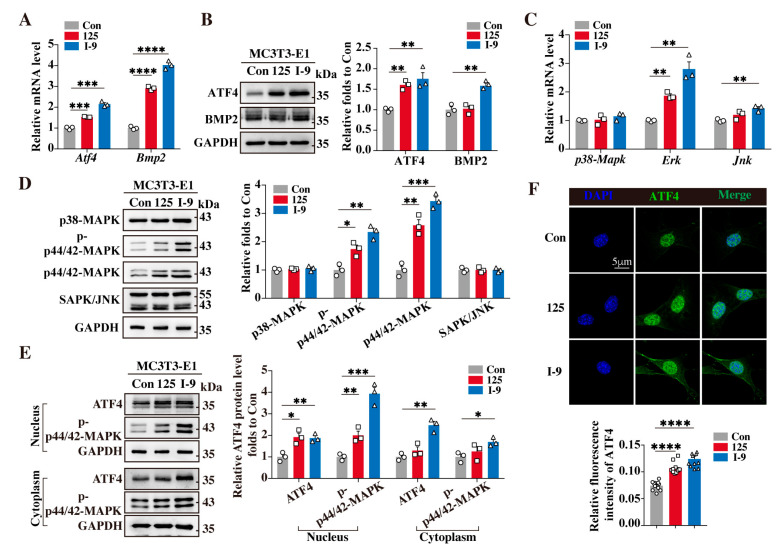
I-9 may upregulate ATF4 expression by activating ERK signaling to promote osteoblast differentiation: (**A**) RT–qPCR analysis of *Atf4* and *Bmp2* transcription in femur tissues from aged mice treated with Con, 125 (5 μM), or I-9 (5 μM) (n = 3); (**B**) Western blot showing ATF4 and BMP2 expression in MC3T3-E1 cells treated with Con, 125 (5 μM), or I-9 (5 μM). Data are representative of three independent experiments; (**C**) RT–qPCR analysis of *p38-Mapk*, *Erk*, and *Jnk* transcription in femur tissues from aged mice treated with Con, 125 (5 μM), or I-9 (5 μM) (n = 3); (**D**) Western blot showing MAPK family expression in MC3T3-E1 cells treated with Con, 125 (5 μM), or I-9 (5 μM). Data are representative of three independent experiments; (**E**) Western blot showing ATF4 and p-p44/42-MAPK expression in the nucleus and cytoplasm of MC3T3-E1 cells treated with Con, 125 (5 μM), or I-9 (5 μM). Data are representative of three independent experiments; (**F**) Immunofluorescence imaging of ATF4 in MC3T3-E1 cells treated with Con, 125 (5 μM), or I-9 (5 μM). Scale bars, 5 µm. Three independent experiments in Con, 125 and I-9 group were indicated by circular symbols, squared symbols and triangular symbols, respectively. Data are presented as the mean ± SEM. Student’s *t*-tests were used to determine statistical significance; * *p* < 0.05, ** *p* < 0.01, *** *p* < 0.001, **** *p* < 0.0001.

We next identified the function of 125 and I-9 in MC3T3-E1 cells. Similar to the in vivo results, 125 enhanced the mRNA level of *Atf4* and *Bmp2*, and the effect was enlarged with I-9 treatment (Figure 6A). Treatment with 125 promoted the ATF4 expression, and I-9 increased the level of ATF4 and BMP2 in MC3T3-E1 cells (Figure 6B). As expected, the mRNA level of *Erk* was increased by treatment with 125 and I-9 (Figure 6C). The phosphorylation of ERK was upregulated by treatment of 125 and I-9 due to the increased total ERK in MC3T3-E1 cells (Figure 6D). ERK activated in endosomes can target nuclear molecules, and therefore, we extracted the cytoplasmic and nuclear components of MC3T3-E1 cells after 125 or I-9 treatment for 24 h. The ATF4 and p-ERK levels were raised in the nucleus and cytoplasm following treatment with I-9 (Figure 6E). In addition, treatment with 125 or I-9 enhanced the fluorescence intensity of ATF4 in MC3T3-E1 cells (Figure 6F). These results indicate that the *Atf4* transcription factor and ERK signaling pathway play an important role in the regulation of benzofuran compounds in osteoporosis. 

### 2.8. Benzofuran Compounds May Promote Osteoblast Differentiation through BMP2 Pathway 

**Figure 7:**
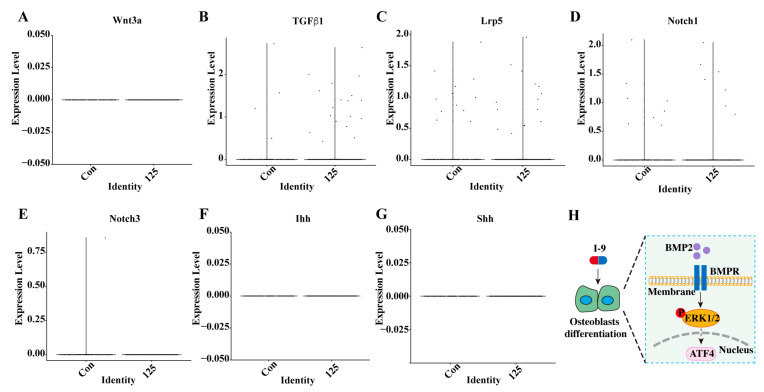
Benzofuran compounds may upregulate osteoblast differentiation through BMP2: (**A**–**G**) Violin plots showing the expression of *Wnt3a*, *Tgfβ1*, *Lrp5*, *Notch1*, *Notch3*, *Ihh*, and *Shh* in osteoblast from Con and 125 groups; (**H**) Schematic shows the mechanism for ATF4 responding to BMP2 activation through the phosphorylation of ERK to promote osteoblast differentiation.

Osteoblast differentiation of bone marrow can be induced by stimulation with Wnt, Notch, and Hedgehog signaling pathways [35,36,37]. To identify the signaling pathway of benzofuran compounds regulating osteoblast differentiation, we analyzed the genes of the Wnt (*Wnt3a, Tgfβ1, Lrp5*), Notch (*Notch1, Notch3*), and Hedgehog (*Ihh, Shh*) signaling pathways in femoral osteoblasts with treatment of 125. And we found that these genes had no significant difference between Con and 125 (Figure 7A–G). Therefore, we consider that benzofuran compounds may upregulate ATF4 expression by improving ERK phosphorylation through BMP2 to promote osteoblast differentiation (Figure 7H).

## 3. Discussion

As the global population increasingly ages, the number of patients with osteoporosis, a highly age-related disease, is rapidly rising. However, patients with osteoporosis pay far less attention to this disease than to tumors, cardiovascular diseases, diabetes, and other diseases. Consequently, many patients with osteoporosis do not receive timely medical treatment, and they continue to lose bone mass and only discover that they have insufficient bone mass at the time of a bone fracture. Once a hip fracture occurs, the rates of disability and mortality within one year are 50% and 20%, respectively. The existing first-line clinical drugs are bone resorption inhibitors, and restoring the bone mass of these patients to the safe range is difficult by inhibiting bone loss. The range and period of use of existing peptides and monoclonal antibodies are limited. Therefore, small-molecule bone formation–promoting drugs are urgently required. We previously found that 6-methoxybenzofuran compound I-9 has the potential as an anti-osteoporotic drug that promotes bone formation. After preliminary pharmacokinetic and toxicological studies, we determined that I-9 can be used as a drug candidate for follow-up studies.

In this study, the synthesis method of I-9 was first investigated to establish a synthesis route with high yield, simple operation, and low cost, which is suitable for future large-scale production. I-9 is obtained by optimizing the structure of the lead compound 125 for the purpose of improving pharmacokinetics. The curative effect of 125 on osteoporosis was confirmed in the early stage of its development, and the efficacy of I-9 was shown to be better than that of 125 and the positive-control drug teriparatide in the animal zebrafish model [29]. Herein, we selected a natural ageing mammalian model to systematically study the efficacy of I-9. Sixteen-month-old C57 mice experience severe bone loss and have a high correlation to patients with low bone mass due to osteoporosis. Micro-CT was used to determine the bone status at the start and end points of I-9 administration in mice. The bone morphology of isolated femur bone was determined via micro-CT, and the number of osteoblasts was evaluated by preparing femur sections. From 16 to 20 months of age, the bone mass of mice in the Con group further decreased, whereas the bone mass of mice in the I-9 group increased, and the amplitude was greater than that in the 125 group. The I-9 group also demonstrated significantly improved bone morphology and increased osteoblast number compared with these parameters in the Con group, suggesting that I-9 could have a significant effect on bone mass improvement in patients with low bone mass osteoporosis.

Due to our previous study, we proposed that the mechanism of 125 and I-9 for the treatment of SOP may be associated with BMP2-induced osteoblast differentiation by upregulating *Atf4* gene expression. To investigate this hypothesis, we performed a series of biological research in vivo and in vitro, and we found that I-9 may upregulate ATF4 expression by increasing the p-ERK protein level to promote osteoblast differentiation. We suggest that I-9 may enhance the expression of BMP2 and promote the binding of BMPR to BMP2, therefore upregulating the p-ERK protein level, but this still needs further research. Obviously, I-9 increased the mRNA and protein levels of ATF4 in the femur, and the protein level may be influenced by transcriptional regulation and post-translational modification. It is known that the liver plays an important role in bone metabolism. I-9 did not increase the ATF4 or BMP2 protein level in liver tissue, but did increase the p-ERK level, indicating that I-9 may be valid for liver tissue through other pathways. Our findings revealed a hitherto undescribed pathway involving the BMP2–ERK–ATF4 axis that promotes osteoblast differentiation.

This study systematically confirmed the efficacy of I-9 in treating osteoporosis, and I-9 demonstrated an excellent safety profile over 4 months of continuous treatment. Furthermore, we clarified the mechanism of action for I-9 promotion of bone formation. These studies further demonstrated the potential value of I-9 for osteoporosis treatment, and we will perform preclinical research on I-9 in the future. Therefore, this study has promoted the development of small-molecule bone formation–promoting drugs, which are expected to improve the clinical treatment of osteoporosis in the future.

## 4. Materials and Methods

### 4.1. Chemistry

All commercially available solvents and reagents were used as received without further purification. All moisture-sensitive reactions were performed under a nitrogen atmosphere in commercially available (J&K Scientific LLC; San Jose, CA, USA) anhydrous solvents. ^1^H and ^13^C NMR spectra were obtained using a Bruker AVANCE 600 (Bruker; San Francisco, CA, USA) spectrometer at 600 MHz, and 151 MHz with TMS as an internal standard. NMR chemical shifts were described in δ (ppm) using residual solvent peaks as standards [DMSO-*d6*, 2.50, 3.31 ppm (^1^H), 39.50 ppm (^13^C)]. All reagents were of analytical grade or chemically pure. Thin-layer chromatography (TLC) was performed on silica gel plates (Merck; Darmstadt, Germany). All reported yields were for isolated products and were not optimized. The purity was determined through high-performance liquid chromatography (HPLC) on an Agilent Technologies instrument of 1260 Infinity II (Agilent, Santa Clara, CA, USA), and all target compounds were confirmed to have >95% purity. Purity was determined by HPLC, and additional structural characterization was performed by proton NMR and carbon NMR, as described below. HPLC conditions were as follows: Agilent C18 LC column 4.6 mm × 150 mm, 4 µm, 10−90% ACN (0.08% TFA) in water (0.08% TFA), 10 min run, flow rate 0.80 mL/min, UV detection (λ = 220, 254 nm). The mass spectra were obtained using liquid chromatography–mass spectrometry (LC-MS) on an LCMS-2020 Shimadzu instrument (Shimadzu; Kyoto City, Japan) using electrospray ionization (ESI).

2-Morpholinoethyl 6-Methoxybenzofuran-2-carboxylate (I-9). Under the protection of nitrogen, anhydrous potassium carbonate (11.0 g, 80.0 mmol, 4.00 equiv) was added to a stirred solution of 2-hydroxy-4-methoxy benzaldehyde (3.0 g, 20.0 mmol, 1.00 equiv) in DMF (100 mL), and ethyl bromoacetate (3.3 g, 20.0 mmol, 1.00 equiv) was dropped at 0 °C and stirred for 1 h. The mixture was then stirred overnight at 90 °C. It was then cooled to room temperature and filtered. The filtrate was diluted with H_2_O (100 mL) and extracted with ethyl acetate (EA, 100 mL × 3). The organic phase was separated, washed with a saturated NaCl solution (100 mL × 3), dried over MgSO_4_, filtered, and concentrated. The residue was purified over a silica gel column (PE: EA = 5:1) to yield ethyl 6-methoxybenzofuran-2-carboxylate (3.5 g, 15.8 mmol, 79% yield) as a white powder. 6-methoxybenzofuran-2-carboxylate (3.5 g, 15.8 mmol, 1.00 equiv) was dissolved in dioxane (50 mL) solution, and then 0.1 N NaOH aqueous solution was added. The mixture was stirred for 3 h at 40 °C, diluted with PE (100 mL), and extracted with H_2_O (50 mL × 3). The aqueous phase was separated, and the pH was adjusted to 3 with 10% hydrochloric acid; a white solid was precipitated, and 6-methoxybenzofuran-2-carboxylic acid (2.7 g, 14.0 mmol, 88% yield) was obtained after filtration and drying. To a stirred solution of 6-methoxybenzofuran-2-carboxylic acid (25.0 g, 0.1 mol, 1.00 equiv) in DCM (100 mL), HOBt (15.0 g, 0.12 mmol, 1.2 equiv) and EDCI (21.0 g, 0.12 mol, 1.2 equiv) were added and stirred for 1 h at room temperature. Then, 2-morpholinoethan-1-amine (14.3 g, 0.11 mol, 1.10 equiv) was added and reacted at room temperature for 16 h, diluted with H_2_O (80 mL), and extracted with EA (100 mL × 3). The organic phase was washed with a saturated NaCl solution (100 mL × 3), dried over anhydrous MgSO_4_, filtered, and concentrated. The residue was purified on a silica gel column (DCM:MeOH = 20:1) to yield I-9 (19.8 g, 65 mmol, 88%) as a white powder. R.T. = 6.6 min, purity 98.78% (analytical HPLC). mp 40–42 °C. ^1^H NMR (600 MHz, DMSO-*d_6_*) *δ* 7.68 (d, *J* = 1.2 Hz, 1H), 7.67 (s, 1H), 7.33 (t, *J* = 1.8 Hz, 1H), 7.00 (dd, *J* = 8.4, 1.8 Hz, 1H), 4.41 (t, *J* = 6.0 Hz, 2H), 3.84 (s, 3H), 3.57 (t, *J* = 4.8 Hz, 4H), 2.68 (t, *J* = 4.8 Hz, 2H), 2.47 (m, 4H); ^13^C NMR (150 MHz, DMSO-*d_6_*) *δ* 160.82, 159.05, 157.10, 144.57, 124.02, 120.23, 115.09, 114.55, 96.37, 66.68, 62.56, 56.95, 56.25, 53.86.

### 4.2. Mouse Models

C57BL/6 mice aged 16 months were purchased from Jiangsu Alingfei Biotechnology Co., LTD. All animal procedures were performed in accordance with the guidelines approved by the Institutional Animal Care and Use Committee (IACUC) of Medicinal Biotechnology, Chinese Academy of Medical Science, and Peking Union Medical College and were approved by the Animal Ethics Committee of the Institute of Medicinal Biotechnology. All the mice were housed in specific pathogen-free facilities under a 12 h light and 12 h dark cycle. Temperature (23 ± 2 °C) and humidity (55%) were maintained during animal housing. All animals were allowed free access to food and water. After adaptive feeding for 1 week, the groups were administered the corresponding drugs. The mice were received pure water (0.1 mL/day, i.g.), 125 (30 mg/kg/day, i.g.), I-9 (30 mg/kg/day, i.g.) for 4 consecutive months, with 1 d of rest after every 6 d of administration. 

### 4.3. Micro-CT Analysis

Before and after 4 months administration, the mice were anesthetized using isoflurane (RWD Life Science Co; Guangdong, China), followed by BMD test using a Quantum FX micro-CT (PerkinElmer; Waltham, MA, USA). The specimens were placed in a cylindrical holder (140 mm in diameter) with the long axis of the bone tissues perpendicular to the X-ray beam and a spatial resolution of 73 mm for the whole body or 40 mm for the femur (55 kV, 114 mA, 4.5 min scan technique time). We selected the third vertebrae as the area of interest. The data were analyzed using the software of the device (Analyze 2.0; PerkinElmer; Waltham, MA, USA) for rodent analysis. The bones of the mice were filled in the software environment, and the bone density of these areas was calculated using the software. 

### 4.4. Bone Histomorphometry

The mice were sacrificed after 4 months of administration. The right femur was taken and the Scanner software of Skyscan1276 Micro-CT (Bruker; San Francisco, CA, USA) was used to scan each sample. The mouse femur was scanned with a voltage of 60 kV, a current of 200 μA, a resolution of 6.5 μm, an exposure time of 650 ms, and a rotation angle of 0.4 degrees. In the bone marrow cavity of the distal femur of mice, 154 layers of bone trabeculae within the bone marrow cavity with a thickness of 1.0 mm were taken as the analysis area of interest (ROI). N-Recon software v2.2 (Bruker; San Francisco, CA, USA) was used for 3D image reconstruction, and CT-AN software v1.10 (Bruker; San Francisco, CA, USA) was used for 3D analysis. Bone volume fraction (BV/TV), trabecular thickness (Tb.Th), trabecular number (Tb.N), trabecular separation (Tb.Sp), structural model index (SMI), and bone mineral density (BMD) were measured in the area of interest.

### 4.5. Hematoxylin and Eosin Staining

All specimens were decalcified for 7 days in 10% EDTA (pH 7.4). Following decalcification, the specimens were dehydrated and embedded in paraffin using TKY-BMB (KeDi; Zhejiang, China). Sections were cut (7 μm thickness) using a microtome (Leica; Hesse, Germany) and stained with hematoxylin and eosin (HE). The bone volume fraction and number of osteoblasts per square millimeter of trabecular bone surface (BS) were calculated using ImageJ software (2.0.0). 

### 4.6. ScRNA-seq Analysis

SeekOne^®^ MM (Microwell & AMP; Magnetic Beads) single-cell transcriptome kit. Total RNA from mouse femurs was extracted using TRIzol reagent. The samples were then subjected to Illumina HiSeq XTen or NovaSeq. Fastp was used to trim primer sequences and low-quality bases of raw reads and to collect basic statistics. Seurat software (v3.0.2) was used for clustering and visualization of the results, and cell type annotation was performed using a method for identifying cell types based on reference datasets provided in the SingleR package. ClusterProfiler software (v3.16.1) was used to perform KEGG pathway enrichment analysis for differentially expressed gene sets in each cluster. Genes were considered significantly differentially expressed if showing ≥1.5-fold change and a *p* value < 0.05. 

### 4.7. Western Blot

Proteins were extracted from aged mice femur or MC3T3-E1 cells with RIPA buffer (Cell Signaling Technology; Danvers, MA, USA). The protein concentrations by BCA protein assay kits (Beyotime Technology; Shanghai, China) were measured. Lysates were resolved by SDS-PAGE, and then proteins were transferred to PVDF membranes for immunoblotting. Tanon 5200 chemiluminescence imaging system (Tanon; Shanghai, China) was used to capture images.

### 4.8. Real-Time PCR

Total RNA was extracted from the femur tissues of mice or MC3T3-E1 cells in each group and then used with the SYBR Premix Ex Taq kit (TaKaRa), following the manufacturer’s instructions. MyCycler thermal cycler and LineGene9600 were used to collect and analyze the data. The primers used are listed in Table 2. 

### 4.9. Immunofluorescence

The MC3T3-E1 cells were fixed in 4% paraformaldehyde for 30 min. And then, 0.5% Triton X-100/PBS was used to permeabilize the cells for 20 min at room temperature. The non-specific antigen sites were blocked by 3% BSA/PBS for 30 min. Then, the slides were incubated with ATF4 antibody (Proteintech; Greater Chicago, IL, USA) overnight at 4 °C, followed by the secondary antibody Alexa Fluor 555 (Thermo Fisher Scientific; Waltham, Ma, USA) for 1 h at room temperature. Using DAPI to counterstain nuclei, the images were captured by confocal fluorescence microscope (Olympus Microsystems; Tokyo, Japan). Imaris 9.3.1 software was used to quantify the images.

### 4.10. Kyoto Encyclopedia of Genes and Genomes (KEGG) Analysis 

A SeekOne MM (Microwell & AMP; Magnetic Beads) single-cell transcriptome kit (seekgene; Beijing, China) extracted total RNAs from the femurs of aged mice and aged 125 mice with TRIzol reagent, and then the samples were tested for Illumina HiSeq XTen or NovaSeq Primer sequences, and low-quality bases of the original reads were cropped using Fastp (0.20.1), and basic statistics were collected. The results were clustered and visualized using Seurat software (3.0.2), and cell-type annotation was performed using a method to identify cell types based on the reference dataset provided in the SingleR package. The KEGG pathway enrichment analysis was performed for the differentially expressed gene sets in each cluster using ClusterProfiler software (3.16.1). Genes were considered significantly differentially expressed if showing a ≥1.5-fold change and a *p* value < 0.05.

### 4.11. Statistical Analysis

All experiments were performed independently at least three times. All quantified data in bar charts with scatter plots are presented as the mean ± SEM. All statistical analyses were performed using GraphPad Prism version 9 software. All quantified data were first tested for conformation to a normal distribution using the Shapiro–Wilk test and were then analyzed by two-tailed Student’s *t*-test (pairwise comparisons) or analysis of variance (ANOVA) followed by Tukey’s post hoc tests (multicomparison), as appropriate. Significance was accepted at the 0.05 level of probability (*p* < 0.05).

## Figures and Tables

**Table 1:** The method and yield of step c in I-9 synthesis ^a^.

Reagent	Solvent	Reaction Condition	Yield (%)
SOCl_2_, anhydrous toluene, Et_3_N	DMF	80 °C–0 °C-rt, 4 h	62%
DCC, DMAP	DMF	rt, 24 h	27%
HATU, Et_3_N	DCM	rt, 24 h	71%
HOBt (1.2 eq), EDCI (1.2 eq)	DCM	rt, 16 h	88%
HOBt (1.1 eq), EDCI (1.1 eq)	DCM	rt, 16 h	79%
HOBt (1.2 eq), EDCI (1.2 eq)	DMF	rt, 16 h	73%
HOBt (1.2 eq), EDCI (1.2 eq)	DCM	45 °C, 16 h	84%
HOBt (1.2 eq), EDCI (1.2 eq)	DCM	rt, 24 h	88%

^a^ SOCl_2_: thionyl chloride; Et_3_N: triethylamine; DMF: dimethyl formamide; rt: room temperature; h: hour; DCC: dicyclohexyl carbodiimide; DMAP: dimethylaminopyridine; eq: equivalent; yield: the ratio between the actual amount of product generated after the reaction of 1 equivalent of 6-methoxybenzofuran-2-carboxylic acid and the theoretical amount of product.

**Table 2:** The primer sequence of real-time PCR.

Gene	Sequence (5′-3′)
Atf4	F: CCTGAACAGCGAAGTGTTGG
	R: TGGAGAACCCATGAGGTTTCAA
Bmp2	F: GGGACCCGCTGTCTTCTAGT
	R: TCAACTCAAATTCGCTGAGGAC
Erk	F: TCCGCCATGAGAATGTTATAGGC
	R: GGTGGTGTTGATAAGCAGATTGG
P38-Mapk	F: TGACCCTTATGACCAGTCCTTT
	R: GTCAGGCTCTTCCACTCATCTAT
Jnk	F: GTGGAATCAAGCACCTTCACT
	R: TCCTCGCCAGTCCAAAATCAA
Gapdh	F: AGGTCGGTGTGAACGGATTTG
	R: GGGGTCGTTGATGGCAACA

F: Forward primer; R: Reverse primer.

## Data Availability

Data are contained within the article.
